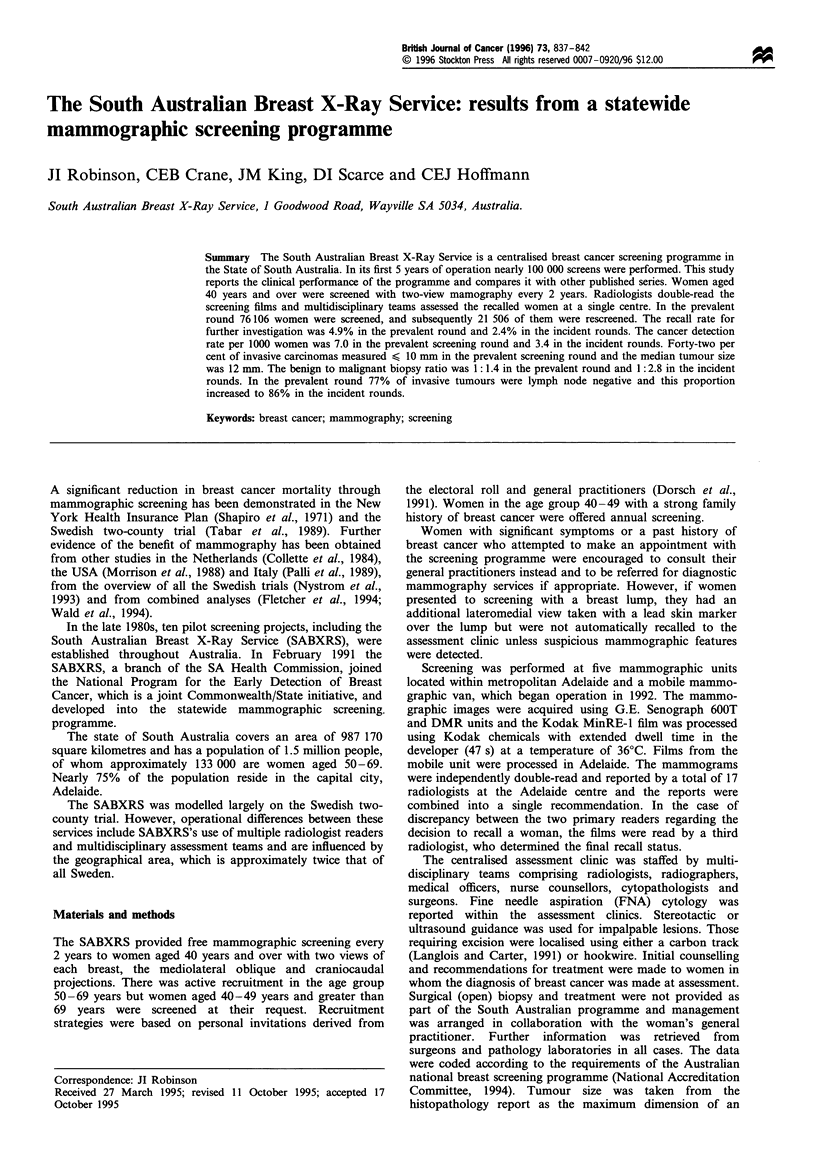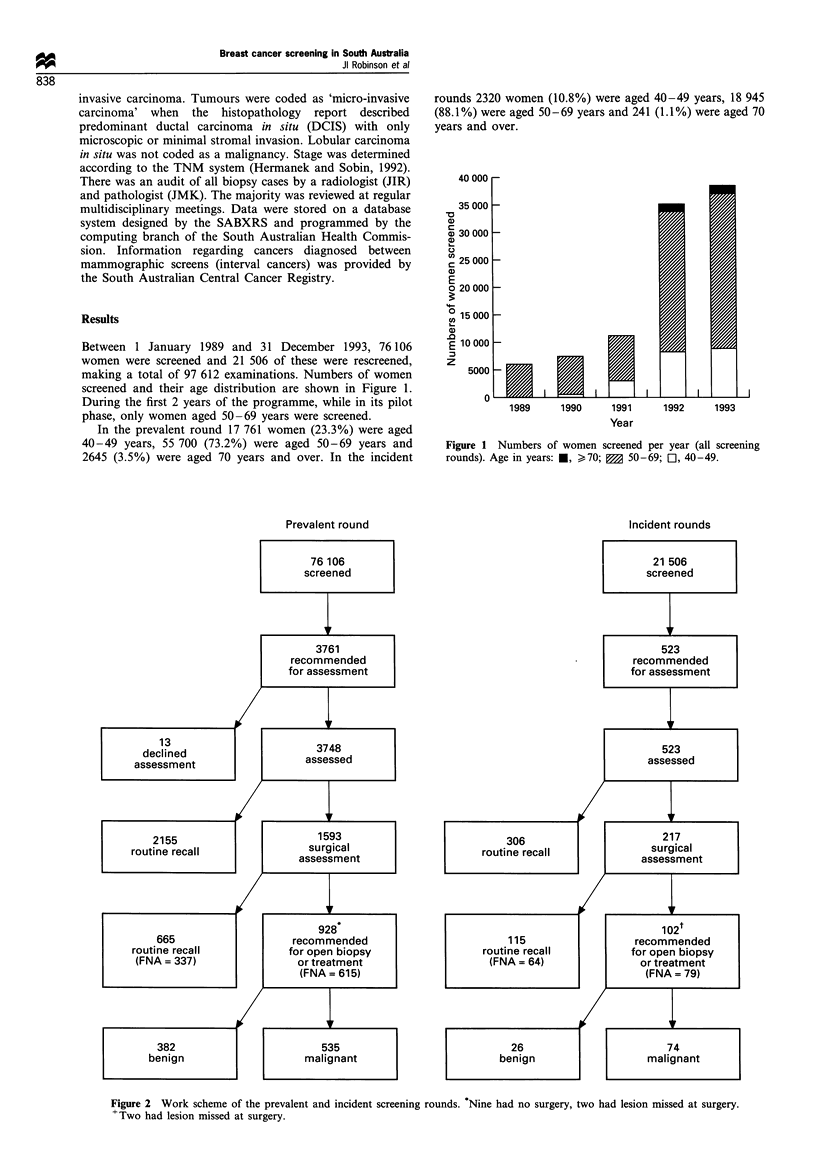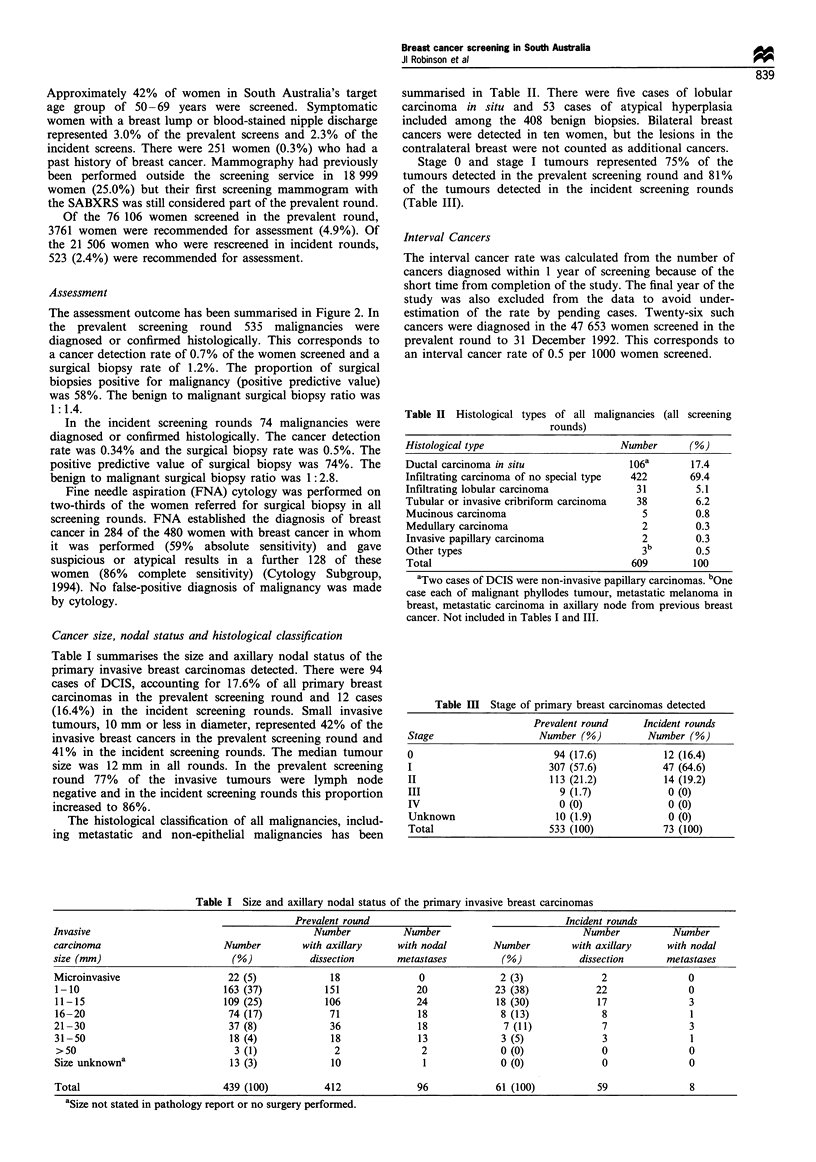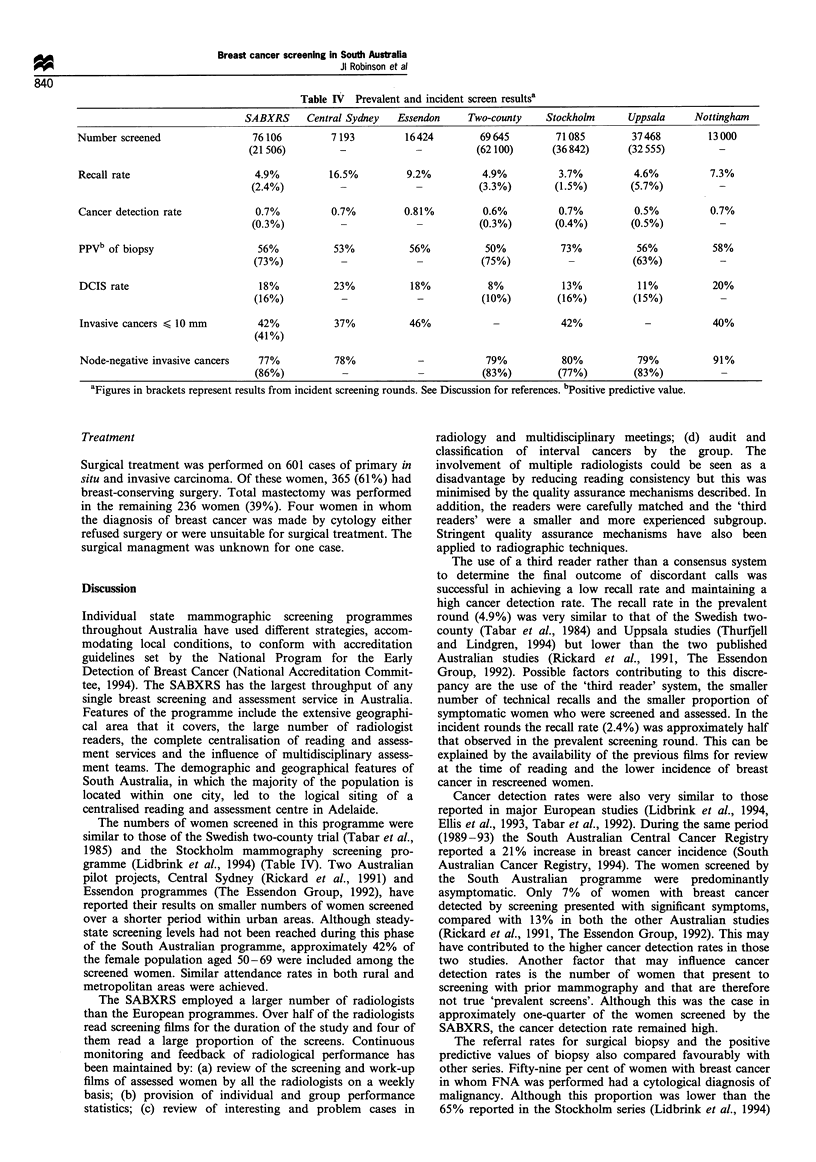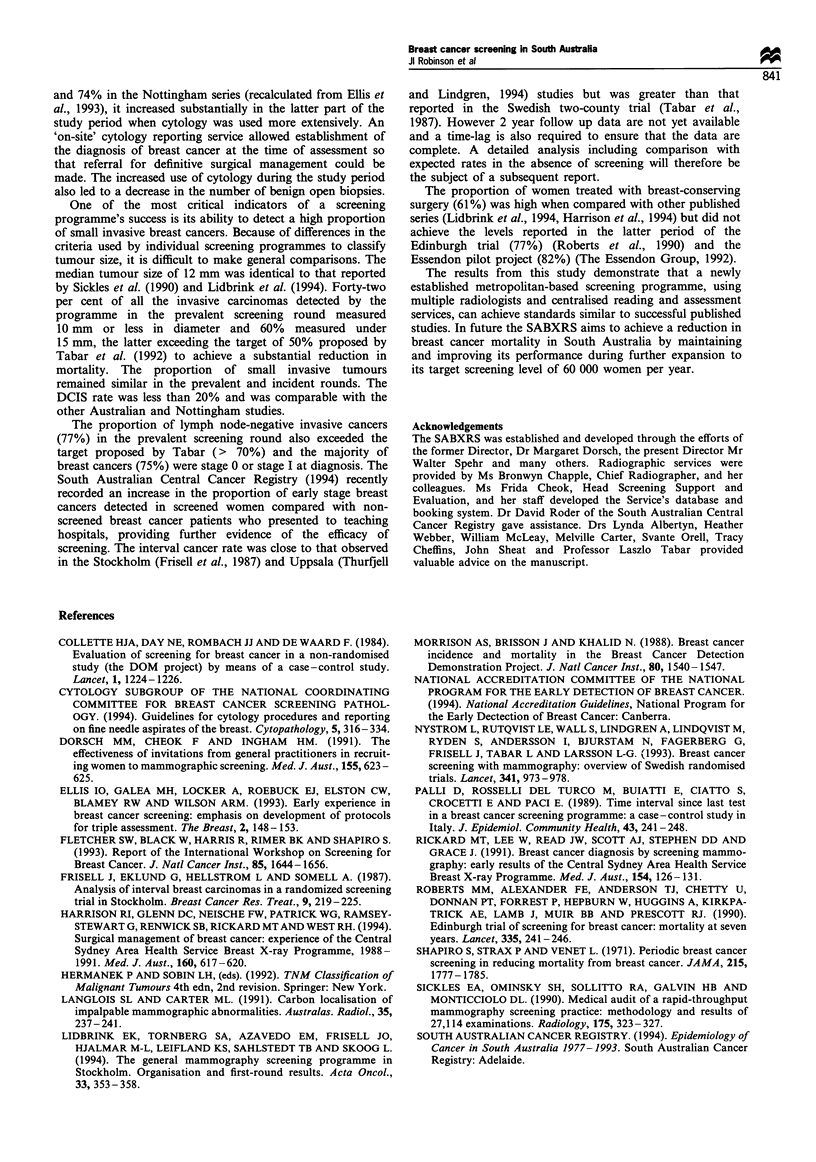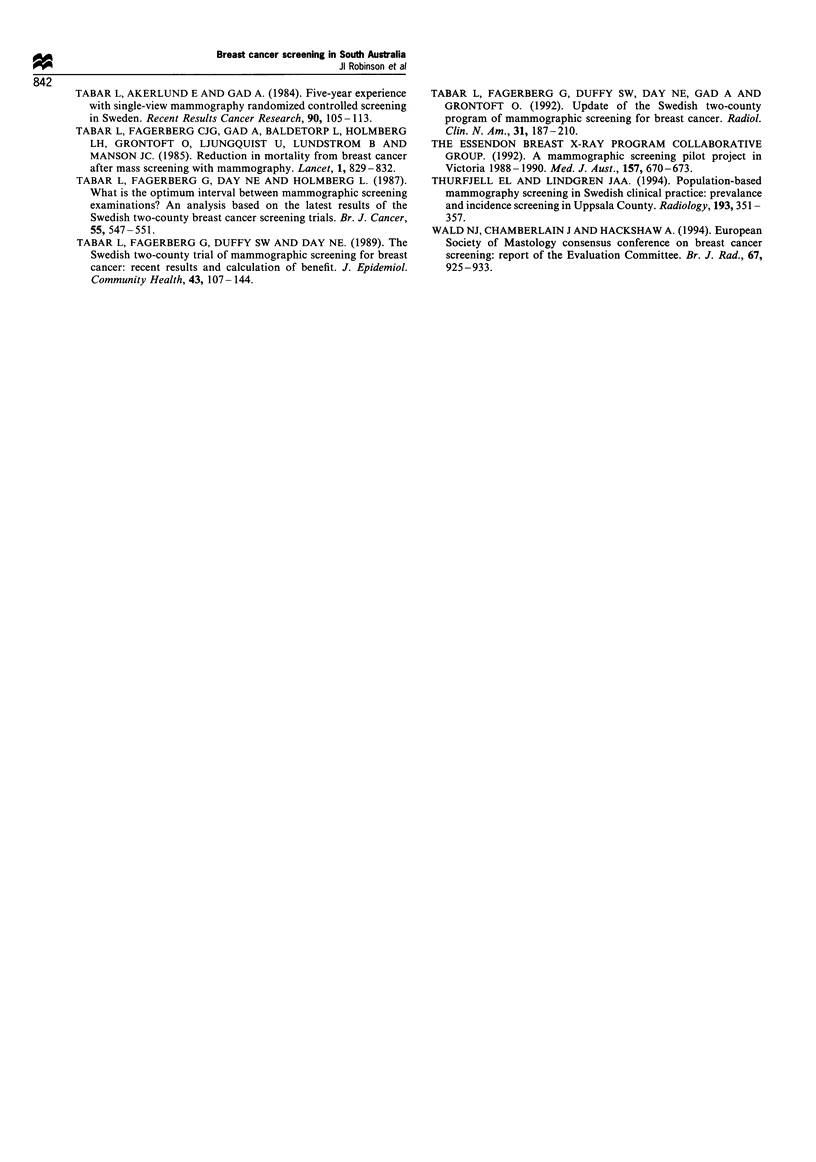# The South Australian Breast X-Ray Service: results from a statewide mammographic screening programme.

**DOI:** 10.1038/bjc.1996.147

**Published:** 1996-03

**Authors:** J. I. Robinson, C. E. Crane, J. M. King, D. I. Scarce, C. E. Hoffmann

**Affiliations:** South Australian Breast X-Ray Service, Wayville, Australia.

## Abstract

The South Australian Breast X-Ray Service is a centralised breast cancer screening programme in the State of South Australia. In its first 5 years of operation nearly 100 000 screens were performed. This study reports the clinical performance of the programme and compares it with other published series. Women aged 40 years and over were screened with two-view mammography every 2 years. Radiologists double-read the screening films and multidisciplinary teams assessed the recalled women at a single centre. In the prevalent round 76 106 women were screened, and subsequently 21 506 of them were rescreened. The recall rate for further investigation was 4.9% in the prevalent round and 2.4% in the incident rounds. The cancer detection rate per 1000 women was 7.0 in the prevalent screening round and 3.4 in the incident rounds. Forty-two per cent of invasive carcinomas measured < or = 10 mm in the prevalent screening round and the median tumour size was 12 mm. The benign to malignant biopsy ratio was 1:1.4 in the prevalent round and 1:2.8 in the incident rounds. In the prevalent round 77% of invasive tumours were lymph node negative and this proportion increased to 86% in the incident rounds.


British Journal of Cancer (1996) 73, 837-842
© 1996 Stockton Press All rights reserved 0007-0920/96 $12.00

### The South Australian Breast X-Ray Service: results from a statewide mammographic screening programme

JI Robinson, CEB Crane, JM King, DI Scarce and CEJ Hoffmann

*South Australian Breast X-Ray Service, 1 Goodwood Road, Wayville SA 5034, Australia.*

**Summary** The South Australian Breast X-Ray Service is a centralised breast cancer screening programme in the State of South Australia. In its first 5 years of operation nearly 100 000 screens were performed. This study reports the clinical performance of the programme and compares it with other published series. Women aged 40 years and over were screened with two-view mamography every 2 years. Radiologists double-read the screening films and multidisciplinary teams assessed the recalled women at a single centre. In the prevalent round 76 106 women were screened, and subsequently 21 506 of them were rescreened. The recall rate for further investigation was 4.9% in the prevalent round and 2.4% in the incident rounds. The cancer detection rate per 1000 women was 7.0 in the prevalent screening round and 3.4 in the incident rounds. Forty-two per cent of invasive carcinomas measured $\leqslant$ 10 mm in the prevalent screening round and the median tumour size was 12 mm. The benign to malignant biopsy ratio was 1 : 1.4 in the prevalent round and 1 : 2.8 in the incident rounds. In the prevalent round 77% of invasive tumours were lymph node negative and this proportion increased to 86% in the incident rounds.

**Keywords:** breast cancer; mammography; screening

A significant reduction in breast cancer mortality through mammographic screening has been demonstrated in the New York Health Insurance Plan (Shapiro *et al.*, 1971) and the Swedish two-county trial (Tabar *et al.*, 1989). Further evidence of the benefit of mammography has been obtained from other studies in the Netherlands (Collette *et al.*, 1984), the USA (Morrison *et al.*, 1988) and Italy (Palli *et al.*, 1989), from the overview of all the Swedish trials (Nystrom *et al.*, 1993) and from combined analyses (Fletcher *et al.*, 1994; Wald *et al.*, 1994).

In the late 1980s, ten pilot screening projects, including the South Australian Breast X-Ray Service (SABXRS), were established throughout Australia. In February 1991 the SABXRS, a branch of the SA Health Commission, joined the National Program for the Early Detection of Breast Cancer, which is a joint Commonwealth/State initiative, and developed into the statewide mammographic screening programme.

The state of South Australia covers an area of 987 170 square kilometres and has a population of 1.5 million people, of whom approximately 133 000 are women aged 50-69. Nearly 75% of the population reside in the capital city, Adelaide.

The SABXRS was modelled largely on the Swedish two-county trial. However, operational differences between these services include SABXRS's use of multiple radiologist readers and multidisciplinary assessment teams and are influenced by the geographical area, which is approximately twice that of all Sweden.

### Materials and methods

The SABXRS provided free mammographic screening every 2 years to women aged 40 years and over with two views of each breast, the mediolateral oblique and craniocaudal projections. There was active recruitment in the age group 50-69 years but women aged 40-49 years and greater than 69 years were screened at their request. Recruitment strategies were based on personal invitations derived from the electoral roll and general practitioners (Dorsch *et al.*, 1991). Women in the age group 40-49 with a strong family history of breast cancer were offered annual screening.

Women with significant symptoms or a past history of breast cancer who attempted to make an appointment with the screening programme were encouraged to consult their general practitioners instead and to be referred for diagnostic mammography services if appropriate. However, if women presented to screening with a breast lump, they had an additional lateromedial view taken with a lead skin marker over the lump but were not automatically recalled to the assessment clinic unless suspicious mammographic features were detected.

Screening was performed at five mammographic units located within metropolitan Adelaide and a mobile mammographic van, which began operation in 1992. The mammographic images were acquired using G.E. Senograph 600T and DMR units and the Kodak MinRE-1 film was processed using Kodak chemicals with extended dwell time in the developer (47 s) at a temperature of 36°C. Films from the mobile unit were processed in Adelaide. The mammograms were independently double-read and reported by a total of 17 radiologists at the Adelaide centre and the reports were combined into a single recommendation. In the case of discrepancy between the two primary readers regarding the decision to recall a woman, the films were read by a third radiologist, who determined the final recall status.

The centralised assessment clinic was staffed by multidisciplinary teams comprising radiologists, radiographers, medical officers, nurse counsellors, cytopathologists and surgeons. Fine needle aspiration (FNA) cytology was reported within the assessment clinics. Stereotactic or ultrasound guidance was used for impalpable lesions. Those requiring excision were localised using either a carbon track (Langlois and Carter, 1991) or hookwire. Initial counselling and recommendations for treatment were made to women in whom the diagnosis of breast cancer was made at assessment. Surgical (open) biopsy and treatment were not provided as part of the South Australian programme and management was arranged in collaboration with the woman's general practitioner. Further information was retrieved from surgeons and pathology laboratories in all cases. The data were coded according to the requirements of the Australian national breast screening programme (National Accreditation Committee, 1994). Tumour size was taken from the histopathology report as the maximum dimension of an

Correspondence: JI Robinson
Received 27 March 1995; revised 11 October 1995; accepted 17 October 1995

invasive carcinoma. Tumours were coded as 'micro-invasive carcinoma' when the histopathology report described predominant ductal carcinoma *in situ* (DCIS) with only microscopic or minimal stromal invasion. Lobular carcinoma *in situ* was not coded as a malignancy. Stage was determined according to the TNM system (Hermanek and Sobin, 1992). There was an audit of all biopsy cases by a radiologist (JIR) and pathologist (JMK). The majority was reviewed at regular multidisciplinary meetings. Data were stored on a database system designed by the SABXRS and programmed by the computing branch of the South Australian Health Commission. Information regarding cancers diagnosed between mammographic screens (interval cancers) was provided by the South Australian Central Cancer Registry.

### Results

Between 1 January 1989 and 31 December 1993, 76 106 women were screened and 21 506 of these were rescreened, making a total of 97 612 examinations. Numbers of women screened and their age distribution are shown in Figure 1. During the first 2 years of the programme, while in its pilot phase, only women aged 50–69 years were screened.

In the prevalent round 17 761 women (23.3%) were aged 40–49 years, 55 700 (73.2%) were aged 50–69 years and 2645 (3.5%) were aged 70 years and over. In the incident rounds 2320 women (10.8%) were aged 40–49 years, 18 945 (88.1%) were aged 50–69 years and 241 (1.1%) were aged 70 years and over.

**Figure 1** Numbers of women screened per year (all screening rounds). Age in years: ■, ⩾70; ▨ 50–69; □, 40–49.

**Figure 2** Work scheme of the prevalent and incident screening rounds. *Nine had no surgery, two had lesion missed at surgery. +Two had lesion missed at surgery.

Approximately 42% of women in South Australia's target age group of 50–69 years were screened. Symptomatic women with a breast lump or blood-stained nipple discharge represented 3.0% of the prevalent screens and 2.3% of the incident screens. There were 251 women (0.3%) who had a past history of breast cancer. Mammography had previously been performed outside the screening service in 18 999 women (25.0%) but their first screening mammogram with the SABXRS was still considered part of the prevalent round.

Of the 76 106 women screened in the prevalent round, 3761 women were recommended for assessment (4.9%). Of the 21 506 women who were rescreened in incident rounds, 523 (2.4%) were recommended for assessment.

### *Assessment*

The assessment outcome has been summarised in Figure 2. In the prevalent screening round 535 malignancies were diagnosed or confirmed histologically. This corresponds to a cancer detection rate of 0.7% of the women screened and a surgical biopsy rate of 1.2%. The proportion of surgical biopsies positive for malignancy (positive predictive value) was 58%. The benign to malignant surgical biopsy ratio was 1 : 1.4.

In the incident screening rounds 74 malignancies were diagnosed or confirmed histologically. The cancer detection rate was 0.34% and the surgical biopsy rate was 0.5%. The positive predictive value of surgical biopsy was 74%. The benign to malignant surgical biopsy ratio was 1 : 2.8.

Fine needle aspiration (FNA) cytology was performed on two-thirds of the women referred for surgical biopsy in all screening rounds. FNA established the diagnosis of breast cancer in 284 of the 480 women with breast cancer in whom it was performed (59% absolute sensitivity) and gave suspicious or atypical results in a further 128 of these women (86% complete sensitivity) (Cytology Subgroup, 1994). No false-positive diagnosis of malignancy was made by cytology.

### *Cancer size, nodal status and histological classification*

Table I summarises the size and axillary nodal status of the primary invasive breast carcinomas detected. There were 94 cases of DCIS, accounting for 17.6% of all primary breast carcinomas in the prevalent screening round and 12 cases (16.4%) in the incident screening rounds. Small invasive tumours, 10 mm or less in diameter, represented 42% of the invasive breast cancers in the prevalent screening round and 41% in the incident screening rounds. The median tumour size was 12 mm in all rounds. In the prevalent screening round 77% of the invasive tumours were lymph node negative and in the incident screening rounds this proportion increased to 86%.

The histological classification of all malignancies, including metastatic and non-epithelial malignancies has been summarised in Table II. There were five cases of lobular carcinoma *in situ* and 53 cases of atypical hyperplasia included among the 408 benign biopsies. Bilateral breast cancers were detected in ten women, but the lesions in the contralateral breast were not counted as additional cancers.

Stage 0 and stage I tumours represented 75% of the tumours detected in the prevalent screening round and 81% of the tumours detected in the incident screening rounds (Table III).

### *Interval Cancers*

The interval cancer rate was calculated from the number of cancers diagnosed within 1 year of screening because of the short time from completion of the study. The final year of the study was also excluded from the data to avoid underestimation of the rate by pending cases. Twenty-six such cancers were diagnosed in the 47 653 women screened in the prevalent round to 31 December 1992. This corresponds to an interval cancer rate of 0.5 per 1000 women screened.

**Table II** Histological types of all malignancies (all screening rounds)

| *Histological type* | *Number* | *(%)* |
|---|---|---|
| Ductal carcinoma *in situ* | 106[a] | 17.4 |
| Infiltrating carcinoma of no special type | 422 | 69.4 |
| Infiltrating lobular carcinoma | 31 | 5.1 |
| Tubular or invasive cribriform carcinoma | 38 | 6.2 |
| Mucinous carcinoma | 5 | 0.8 |
| Medullary carcinoma | 2 | 0.3 |
| Invasive papillary carcinoma | 2 | 0.3 |
| Other types | 3[b] | 0.5 |
| Total | 609 | 100 |

[a]Two cases of DCIS were non-invasive papillary carcinomas. [b]One case each of malignant phyllodes tumour, metastatic melanoma in breast, metastatic carcinoma in axillary node from previous breast cancer. Not included in Tables I and III.

**Table III** Stage of primary breast carcinomas detected

| *Stage* | *Prevalent round Number (%)* | *Incident rounds Number (%)* |
|---|---|---|
| 0 | 94 (17.6) | 12 (16.4) |
| I | 307 (57.6) | 47 (64.6) |
| II | 113 (21.2) | 14 (19.2) |
| III | 9 (1.7) | 0 (0) |
| IV | 0 (0) | 0 (0) |
| Unknown | 10 (1.9) | 0 (0) |
| Total | 533 (100) | 73 (100) |

**Table I** Size and axillary nodal status of the primary invasive breast carcinomas

| *Invasive carcinoma size (mm)* | *Prevalent round* Number (%) | Number with axillary dissection | Number with nodal metastases | *Incident rounds* Number (%) | Number with axillary dissection | Number with nodal metastases |
|---|---|---|---|---|---|---|
| Microinvasive | 22 (5) | 18 | 0 | 2 (3) | 2 | 0 |
| 1–10 | 163 (37) | 151 | 20 | 23 (38) | 22 | 0 |
| 11–15 | 109 (25) | 106 | 24 | 18 (30) | 17 | 3 |
| 16–20 | 74 (17) | 71 | 18 | 8 (13) | 8 | 1 |
| 21–30 | 37 (8) | 36 | 18 | 7 (11) | 7 | 3 |
| 31–50 | 18 (4) | 18 | 13 | 3 (5) | 3 | 1 |
| >50 | 3 (1) | 2 | 2 | 0 (0) | 0 | 0 |
| Size unknown[a] | 13 (3) | 10 | 1 | 0 (0) | 0 | 0 |
| Total | 439 (100) | 412 | 96 | 61 (100) | 59 | 8 |

[a]Size not stated in pathology report or no surgery performed.

Table IV Prevalent and incident screen results[a]

| | *SABXRS* | *Central Sydney* | *Essendon* | *Two-county* | *Stockholm* | *Uppsala* | *Nottingham* |
|---|---|---|---|---|---|---|---|
| Number screened | 76 106 (21 506) | 7 193 – | 16 424 – | 69 645 (62 100) | 71 085 (36 842) | 37 468 (32 555) | 13 000 – |
| Recall rate | 4.9% (2.4%) | 16.5% – | 9.2% – | 4.9% (3.3%) | 3.7% (1.5%) | 4.6% (5.7%) | 7.3% – |
| Cancer detection rate | 0.7% (0.3%) | 0.7% – | 0.81% – | 0.6% (0.3%) | 0.7% (0.4%) | 0.5% (0.5%) | 0.7% – |
| PPV[b] of biopsy | 56% (73%) | 53% – | 56% – | 50% (75%) | 73% – | 56% (63%) | 58% – |
| DCIS rate | 18% (16%) | 23% – | 18% – | 8% (10%) | 13% (16%) | 11% (15%) | 20% – |
| Invasive cancers ⩽ 10 mm | 42% (41%) | 37% | 46% | – | 42% | – | 40% |
| Node-negative invasive cancers | 77% (86%) | 78% – | – – | 79% (83%) | 80% (77%) | 79% (83%) | 91% – |

[a]Figures in brackets represent results from incident screening rounds. See Discussion for references. [b]Positive predictive value.

*Treatment*

Surgical treatment was performed on 601 cases of primary *in situ* and invasive carcinoma. Of these women, 365 (61%) had breast-conserving surgery. Total mastectomy was performed in the remaining 236 women (39%). Four women in whom the diagnosis of breast cancer was made by cytology either refused surgery or were unsuitable for surgical treatment. The surgical managment was unknown for one case.

### Discussion

Individual state mammographic screening programmes throughout Australia have used different strategies, accommodating local conditions, to conform with accreditation guidelines set by the National Program for the Early Detection of Breast Cancer (National Accreditation Committee, 1994). The SABXRS has the largest throughput of any single breast screening and assessment service in Australia. Features of the programme include the extensive geographical area that it covers, the large number of radiologist readers, the complete centralisation of reading and assessment services and the influence of multidisciplinary assessment teams. The demographic and geographical features of South Australia, in which the majority of the population is located within one city, led to the logical siting of a centralised reading and assessment centre in Adelaide.

The numbers of women screened in this programme were similar to those of the Swedish two-county trial (Tabar *et al.*, 1985) and the Stockholm mammography screening programme (Lidbrink *et al.*, 1994) (Table IV). Two Australian pilot projects, Central Sydney (Rickard *et al.*, 1991) and Essendon programmes (The Essendon Group, 1992), have reported their results on smaller numbers of women screened over a shorter period within urban areas. Although steady-state screening levels had not been reached during this phase of the South Australian programme, approximately 42% of the female population aged 50–69 were included among the screened women. Similar attendance rates in both rural and metropolitan areas were achieved.

The SABXRS employed a larger number of radiologists than the European programmes. Over half of the radiologists read screening films for the duration of the study and four of them read a large proportion of the screens. Continuous monitoring and feedback of radiological performance has been maintained by: (a) review of the screening and work-up films of assessed women by all the radiologists on a weekly basis; (b) provision of individual and group performance statistics; (c) review of interesting and problem cases in radiology and multidisciplinary meetings; (d) audit and classification of interval cancers by the group. The involvement of multiple radiologists could be seen as a disadvantage by reducing reading consistency but this was minimised by the quality assurance mechanisms described. In addition, the readers were carefully matched and the 'third readers' were a smaller and more experienced subgroup. Stringent quality assurance mechanisms have also been applied to radiographic techniques.

The use of a third reader rather than a consensus system to determine the final outcome of discordant calls was successful in achieving a low recall rate and maintaining a high cancer detection rate. The recall rate in the prevalent round (4.9%) was very similar to that of the Swedish two-county (Tabar *et al.*, 1984) and Uppsala studies (Thurfjell and Lindgren, 1994) but lower than the two published Australian studies (Rickard *et al.*, 1991, The Essendon Group, 1992). Possible factors contributing to this discrepancy are the use of the 'third reader' system, the smaller number of technical recalls and the smaller proportion of symptomatic women who were screened and assessed. In the incident rounds the recall rate (2.4%) was approximately half that observed in the prevalent screening round. This can be explained by the availability of the previous films for review at the time of reading and the lower incidence of breast cancer in rescreened women.

Cancer detection rates were also very similar to those reported in major European studies (Lidbrink *et al.*, 1994, Ellis *et al.*, 1993, Tabar *et al.*, 1992). During the same period (1989–93) the South Australian Central Cancer Registry reported a 21% increase in breast cancer incidence (South Australian Cancer Registry, 1994). The women screened by the South Australian programme were predominantly asymptomatic. Only 7% of women with breast cancer detected by screening presented with significant symptoms, compared with 13% in both the other Australian studies (Rickard *et al.*, 1991, The Essendon Group, 1992). This may have contributed to the higher cancer detection rates in those two studies. Another factor that may influence cancer detection rates is the number of women that present to screening with prior mammography and that are therefore not true 'prevalent screens'. Although this was the case in approximately one-quarter of the women screened by the SABXRS, the cancer detection rate remained high.

The referral rates for surgical biopsy and the positive predictive values of biopsy also compared favourably with other series. Fifty-nine per cent of women with breast cancer in whom FNA was performed had a cytological diagnosis of malignancy. Although this proportion was lower than the 65% reported in the Stockholm series (Lidbrink *et al.*, 1994)

and 74% in the Nottingham series (recalculated from Ellis et al., 1993), it increased substantially in the latter part of the study period when cytology was used more extensively. An 'on-site' cytology reporting service allowed establishment of the diagnosis of breast cancer at the time of assessment so that referral for definitive surgical management could be made. The increased use of cytology during the study period also led to a decrease in the number of benign open biopsies.

One of the most critical indicators of a screening programme's success is its ability to detect a high proportion of small invasive breast cancers. Because of differences in the criteria used by individual screening programmes to classify tumour size, it is difficult to make general comparisons. The median tumour size of 12 mm was identical to that reported by Sickles et al. (1990) and Lidbrink et al. (1994). Forty-two per cent of all the invasive carcinomas detected by the programme in the prevalent screening round measured 10 mm or less in diameter and 60% measured under 15 mm, the latter exceeding the target of 50% proposed by Tabar et al. (1992) to achieve a substantial reduction in mortality. The proportion of small invasive tumours remained similar in the prevalent and incident rounds. The DCIS rate was less than 20% and was comparable with the other Australian and Nottingham studies.

The proportion of lymph node-negative invasive cancers (77%) in the prevalent screening round also exceeded the target proposed by Tabar (> 70%) and the majority of breast cancers (75%) were stage 0 or stage I at diagnosis. The South Australian Central Cancer Registry (1994) recently recorded an increase in the proportion of early stage breast cancers detected in screened women compared with non-screened breast cancer patients who presented to teaching hospitals, providing further evidence of the efficacy of screening. The interval cancer rate was close to that observed in the Stockholm (Frisell et al., 1987) and Uppsala (Thurfjell and Lindgren, 1994) studies but was greater than that reported in the Swedish two-county trial (Tabar et al., 1987). However 2 year follow up data are not yet available and a time-lag is also required to ensure that the data are complete. A detailed analysis including comparison with expected rates in the absence of screening will therefore be the subject of a subsequent report.

The proportion of women treated with breast-conserving surgery (61%) was high when compared with other published series (Lidbrink et al., 1994, Harrison et al., 1994) but did not achieve the levels reported in the latter period of the Edinburgh trial (77%) (Roberts et al., 1990) and the Essendon pilot project (82%) (The Essendon Group, 1992).

The results from this study demonstrate that a newly established metropolitan-based screening programme, using multiple radiologists and centralised reading and assessment services, can achieve standards similar to successful published studies. In future the SABXRS aims to achieve a reduction in breast cancer mortality in South Australia by maintaining and improving its performance during further expansion to its target screening level of 60 000 women per year.

**Acknowledgements**
The SABXRS was established and developed through the efforts of the former Director, Dr Margaret Dorsch, the present Director Mr Walter Spehr and many others. Radiographic services were provided by Ms Bronwyn Chapple, Chief Radiographer, and her colleagues. Ms Frida Cheok, Head Screening Support and Evaluation, and her staff developed the Service's database and booking system. Dr David Roder of the South Australian Central Cancer Registry gave assistance. Drs Lynda Albertyn, Heather Webber, William McLeay, Melville Carter, Svante Orell, Tracy Cheffins, John Sheat and Professor Laszlo Tabar provided valuable advice on the manuscript.

### References

COLLETTE HJA, DAY NE, ROMBACH JJ AND DE WAARD F. (1984). Evaluation of screening for breast cancer in a non-randomised study (the DOM project) by means of a case-control study. *Lancet*, **1**, 1224-1226.

CYTOLOGY SUBGROUP OF THE NATIONAL COORDINATING COMMITTEE FOR BREAST CANCER SCREENING PATHOLOGY. (1994). Guidelines for cytology procedures and reporting on fine needle aspirates of the breast. *Cytopathology*, **5**, 316-334.

DORSCH MM, CHEOK F AND INGHAM HM. (1991). The effectiveness of invitations from general practitioners in recruiting women to mammographic screening. *Med. J. Aust.*, **155**, 623-625.

ELLIS IO, GALEA MH, LOCKER A, ROEBUCK EJ, ELSTON CW, BLAMEY RW AND WILSON ARM. (1993). Early experience in breast cancer screening: emphasis on development of protocols for triple assessment. *The Breast*, **2**, 148-153.

FLETCHER SW, BLACK W, HARRIS R, RIMER BK AND SHAPIRO S. (1993). Report of the International Workshop on Screening for Breast Cancer. *J. Natl Cancer Inst.*, **85**, 1644-1656.

FRISELL J, EKLUND G, HELLSTROM L AND SOMELL A. (1987). Analysis of interval breast carcinomas in a randomized screening trial in Stockholm. *Breast Cancer Res. Treat.*, **9**, 219-225.

HARRISON RI, GLENN DC, NEISCHE FW, PATRICK WG, RAMSEY-STEWART G, RENWICK SB, RICKARD MT AND WEST RH. (1994). Surgical management of breast cancer: experience of the Central Sydney Area Health Service Breast X-ray Programme, 1988-1991. *Med. J. Aust.*, **160**, 617-620.

HERMANEK P AND SOBIN LH, (eds). (1992). *TNM Classification of Malignant Tumours* 4th edn, 2nd revision. Springer: New York.

LANGLOIS SL AND CARTER ML. (1991). Carbon localisation of impalpable mammographic abnormalities. *Australas. Radiol.*, **35**, 237-241.

LIDBRINK EK, TORNBERG SA, AZAVEDO EM, FRISELL JO, HJALMAR M-L, LEIFLAND KS, SAHLSTEDT TB AND SKOOG L. (1994). The general mammography screening programme in Stockholm. Organisation and first-round results. *Acta Oncol.*, **33**, 353-358.

MORRISON AS, BRISSON J AND KHALID N. (1988). Breast cancer incidence and mortality in the Breast Cancer Detection Demonstration Project. *J. Natl Cancer Inst.*, **80**, 1540-1547.

NATIONAL ACCREDITATION COMMITTEE OF THE NATIONAL PROGRAM FOR THE EARLY DETECTION OF BREAST CANCER. (1994). *National Accreditation Guidelines*, National Program for the Early Dectection of Breast Cancer: Canberra.

NYSTROM L, RUTQVIST LE, WALL S, LINDGREN A, LINDQVIST M, RYDEN S, ANDERSSON I, BJURSTAM N, FAGERBERG G, FRISELL J, TABAR L AND LARSSON L-G. (1993). Breast cancer screening with mammography: overview of Swedish randomised trials. *Lancet*, **341**, 973-978.

PALLI D, ROSSELLI DEL TURCO M, BUIATTI E, CIATTO S, CROCETTI E AND PACI E. (1989). Time interval since last test in a breast cancer screening programme: a case-control study in Italy. *J. Epidemiol. Community Health*, **43**, 241-248.

RICKARD MT, LEE W, READ JW, SCOTT AJ, STEPHEN DD AND GRACE J. (1991). Breast cancer diagnosis by screening mammography: early results of the Central Sydney Area Health Service Breast X-ray Programme. *Med. J. Aust.*, **154**, 126-131.

ROBERTS MM, ALEXANDER FE, ANDERSON TJ, CHETTY U, DONNAN PT, FORREST P, HEPBURN W, HUGGINS A, KIRKPATRICK AE, LAMB J, MUIR BB AND PRESCOTT RJ. (1990). Edinburgh trial of screening for breast cancer: mortality at seven years. *Lancet*, **335**, 241-246.

SHAPIRO S, STRAX P AND VENET L. (1971). Periodic breast cancer screening in reducing mortality from breast cancer. *JAMA*, **215**, 1777-1785.

SICKLES EA, OMINSKY SH, SOLLITTO RA, GALVIN HB AND MONTICCIOLO DL. (1990). Medical audit of a rapid-throughput mammography screening practice: methodology and results of 27,114 examinations. *Radiology*, **175**, 323-327.

SOUTH AUSTRALIAN CANCER REGISTRY. (1994). *Epidemiology of Cancer in South Australia 1977-1993*. South Australian Cancer Registry: Adelaide.

TABAR L, AKERLUND E AND GAD A. (1984). Five-year experience with single-view mammography randomized controlled screening in Sweden. *Recent Results Cancer Research*, **90,** 105–113.

TABAR L, FAGERBERG CJG, GAD A, BALDETORP L, HOLMBERG LH, GRONTOFT O, LJUNGQUIST U, LUNDSTROM B AND MANSON JC. (1985). Reduction in mortality from breast cancer after mass screening with mammography. *Lancet*, **1,** 829–832.

TABAR L, FAGERBERG G, DAY NE AND HOLMBERG L. (1987). What is the optimum interval between mammographic screening examinations? An analysis based on the latest results of the Swedish two-county breast cancer screening trials. *Br. J. Cancer*, **55,** 547–551.

TABAR L, FAGERBERG G, DUFFY SW AND DAY NE. (1989). The Swedish two-county trial of mammographic screening for breast cancer: recent results and calculation of benefit. *J. Epidemiol. Community Health*, **43,** 107–144.

TABAR L, FAGERBERG G, DUFFY SW, DAY NE, GAD A AND GRONTOFT O. (1992). Update of the Swedish two-county program of mammographic screening for breast cancer. *Radiol. Clin. N. Am.*, **31,** 187–210.

THE ESSENDON BREAST X-RAY PROGRAM COLLABORATIVE GROUP. (1992). A mammographic screening pilot project in Victoria 1988–1990. *Med. J. Aust.*, **157,** 670–673.

THURFJELL EL AND LINDGREN JAA. (1994). Population-based mammography screening in Swedish clinical practice: prevalance and incidence screening in Uppsala County. *Radiology*, **193,** 351–357.

WALD NJ, CHAMBERLAIN J AND HACKSHAW A. (1994). European Society of Mastology consensus conference on breast cancer screening: report of the Evaluation Committee. *Br. J. Rad.*, **67,** 925–933.